# Perceived Coach Leadership Profiles and Relationship With Burnout, Coping, and Emotions

**DOI:** 10.3389/fpsyg.2019.01785

**Published:** 2019-08-13

**Authors:** Higinio González-García, Guillaume Martinent, Alfonso Trinidad Morales

**Affiliations:** ^1^Faculty of Education, International University of La Rioja, La Rioja, Spain; ^2^Laboratory of Vulnerabilities and Innovation in Sport (EA 7428), Interdisciplinary Research Confederation in Sport (FED 4272), University of Claude Bernard Lyon 1, Univ Lyon, Lyon, France; ^3^Faculty of Education and Humanities, Francisco de Vitoria University, Madrid, Spain

**Keywords:** athlete burnout, cluster analysis, coping, coach leadership, emotion

## Abstract

The aims of the study were to identify coach profiles and examine whether participants from distinct profiles significantly differed on burnout, emotions, and coping or not. A sample of 268 athletes (*M*_*age* = _ 29.34; *SD* = 12.37) completed a series of self-reported questionnaires. Cluster analyses revealed two coach leadership profiles: (a) profile 1 with high scores of training and instruction, authoritarian behavior, social support, and positive feedback, and a low score of democratic behavior; and (b) profile 2 with low levels in training and instruction, authoritarian behavior, social support, and positive feedback and high levels in democratic behavior. Results of Multivariate analyses of variance (MANOVAs) indicated significant differences across coach profiles on reduced accomplishment, sport devaluation, happiness and seeking support and marginal differences on dejection, logical analysis, imagery/thought control, and excitement. Moreover, coach leadership profiles were not confounded by demographic variables (level of competition, gender, age, number of practice hours, professional versus no professional athletes). As a conclusion, the profile approach offered a holistic way to examine coach leadership in sport as two distinct coach profiles emerged from the cluster analyses with an unexpected combination of coach leadership dimensions.

## Introduction

Coach leadership in sporting contexts has been a widely studied topic during the last three decades (Chelladurai and Saleh, 1980; Baker et al., 2000; Cruz and Kim, 2017; Ekstrand et al., 2017). Result of this literature has shown that coach leadership is related to a wide range of positive and negative athlete outcomes such as burnout, coping, satisfaction with sports practice, emotions, sport performance, collective efficacy, or injuries (Baker et al., 2000; Cruz and Kim, 2017; Ekstrand et al., 2017). Chelladurai’s Multidimensional Model (1980) has been the most widely used in the sport field. This model suggests that sport performance and satisfaction of the training group members depends on the congruence among required, current, and preferred coach leadership style (Fletcher and Roberts, 2013). In this model, coach leadership is a multidimensional construct comprising the dimensions of democratic, autocratic, training and instruction, social support, and positive feedback (Chelladurai and Saleh, 1980).

Concerning coach sport leadership studies, the literature has revealed that coaches perceived as giving more training and instructions, social support, and positive feedback resulted in higher levels of athletes’ sport participation, self-determined motivation, fun, group cohesion, and lower levels of anxiety and burnout (Hollembeak and Amorose, 2005; Sullah et al., 2014; Cruz and Kim, 2017). Positive feedback was related to athletes’ perceived ability, the relationship between athletes and coaches, athlete’s efforts, and performance (Hollembeak and Amorose, 2005; Chia et al., 2015; Ignacio et al., 2017). In contrast, coach autocratic behavior was negatively related to the relationship between coaches and athletes (Amorose and Smith, 2003; Ignacio et al., 2017).

Literature provided evidence that there were several studies that examined preferred leadership by athletes relating it to gender, athlete’s satisfaction, type of sport, and level of competition (Witte, 2011; Chia et al., 2015; Cruz and Kim, 2017; Ignacio et al., 2017). Regarding the sport type, Witte (2011) provided evidence that the type of coach leadership, preferred by individual sports athletes, should be led by positive feedback, democratic behavior, training and instruction, contextual considerations, and social support. According to that, in one study in football, it was shown that the highest league football players and the lowers, in terms of league division, preferred a more democratic coach in both cases (Sullah et al., 2014). Concerning athlete’s satisfaction, Ignacio et al. (2017) highlighted that coaches perceived as more kind on training and instruction, giving recognition, rewards, and positive feedback and socially supporters, increase satisfaction in athletes. In terms of gender, Cruz and Kim (2017) found that: (a) male athletes with female coaches preferred more democratic behavior, autocratic behavior, and social support than those with male coaches; and (b) female athletes with male coaches preferred more democratic behavior, autocratic behavior, and social support than those with female coaches. Furthermore, Sullah et al. (2014) revealed differences on gender in the leadership perception, for example, male athletes perceived their coach leadership behaviors exhibited more on training behavior, democratic behavior, rewarding behavior, social support, and autocratic behavior. While female athletes perceived their coach leadership behaviors exhibited more on training behavior, social support, rewarding behavior, democratic behavior, and autocratic behavior.

Athletes are influenced by coaches in many situations, such as training, competition or social relationship (Jowett and Cockerill, 2003; Hollembeak and Amorose, 2005; Dell’Antonio and Couto, 2014; Ignacio et al., 2017). For instance, coach leadership style is linked with the emotions, experienced by athletes during sport competition (Jowett and Lavallee, 2007; Kristiansen et al., 2008). Emotions can be conceptualized as organized psychophysiological reactions (subjective experience, facial expression, cognitive processing, physiological changes) to ongoing relationships with the environment, reflecting the transaction between a person and its environment (Lazarus, 2000). In the context of sports, athletes experiment a variety of pleasant and unpleasant emotions, which require the ability to regulate them properly (Jones et al., 2005; Martinent et al., 2013, 2018). Hence, due to the negative consequences of the lack of emotional control in sports, it is needed that coaches lead athletes to detect and cope with these emotions (Wagstaff et al., 2012; Laborde et al., 2016). Coping refers to the set of cognitive and behavioral efforts, developed by individuals to control the several internal and/or external demands evaluated as exceeding their perceived resources (Lazarus and Folkman, 1984). Nicolas et al. (2011) indicated that supportive coaching behavior was positively linked with task oriented coping whereas unsupportive coaching behavior was a positive predictor of disengagement oriented coping. Supportive coaching behavior has also shown positive relationships with athlete’s mental skills (Ntoumanis et al., 1999; Sullah et al., 2014). A recent study also showed negative relationships between positive supportive coaching behavior with both challenge and threat appraisals whereas unsupportive coaching behaviors were positively associated with threat appraisal (Levy et al., 2016). Hence, a strong coach–athlete relationship could have negative consequences (Levy et al., 2016). Finally, negative coach behaviors such as manipulating, favoritism, threatening, intimidating, yelling, and upsetting were linked with athlete’s pressure from coaches, ego motivational climate, and disengagement oriented coping (Kristiansen et al., 2008).

Another important variable that can affect athletes sports career, is burnout, which can be defined as a syndrome characterized by emotional and physical exhaustion, sport devaluation, and reduced sense of accomplishment (Raedeke, 1997; Raedeke and Smith, 2001). Emotional and physical exhaustion is produced by the feeling of tiredness due to the high demands in sport competition and a low personal accomplishment (Raedeke and Smith, 2001). Sport devaluation is considered as the loss of interest in sports with a progressive desire of withdrawal (Raedeke, 1997). Reduced sense of accomplishment is defined as the lack of success feeling and self-growth in sports context (Raedeke and Smith, 2001). Sports literature suggested that athlete burnout is linked with the treatment received by athletes from coaches (Frazer-Thomas et al., 2005; Stebbings et al., 2012). In particular, several studies provided evidence of the influence of an authoritarian leadership on burnout syndrome (Ryan and Deci, 2000; Gillet et al., 2010). In contrast, democratic behavior from coaches, social support, autonomy, and positive feedback were positively related to psychological well being in athletes (Sunar et al., 2009; Zardoshtian et al., 2012; DeFreese and Smith, 2013).

As reviewed in the precedent paragraphs, previous studies have mainly investigated the bivariate relationships between the dimensions of coach leaderships and some other variables such as emotion or burnout (see for a review Chelladurai, 1990; Jowett and Cockerill, 2003). Hence, the multivariate nature of this construct has generally been neglected. However, the several dimensions of coach leadership could operate in conjunction with one another. Thus, the impact of a particular dimension of coach leadership might vary as a function of the other one. Many information might be lost if coach leadership dimensions are examined discretely and in isolation of one another, as this does not encompass the systemic nature of the construct (i.e., interplay among coach leadership dimensions). In order to explore the multivariate nature of the coach leadership construct, the present study adopted a coach leadership profile approach. This approach could provide a meaningful way to summarize the different coach leadership dimensions. Until the date, no study adopted a methodological profile approach for examining the construct of coach leadership in sports settings. Examination of coach leadership profiles could further our understanding of how the several dimensions of this construct may operate. In turn, this could help sports psychologist adapting their intervention according to the needs of specific groups of athletes. Therefore, the goals of the study were to identify coach leadership profiles and examine whether participants from distinct profiles significantly differed on burnout, emotions and coping. Given that few studies were grounded in a coach leadership profile, no specific hypotheses were advanced on the number of profiles or their characteristics. Concerning the relationships between coach leadership profiles, emotion, burnout and coping, in line with empirical research (Gillet et al., 2010; Zardoshtian et al., 2012; DeFreese and Smith, 2013), we hypothesized that: (a) coach leadership profiles, characterized by high scores of positive feedback, coach democratic behavior, social support and training, an instruction will report higher scores of coping, pleasant emotions (excitement, happiness) and lower scores of burnout; (b) coach leadership profiles, characterized by high scores of, logical analysis, imagery/thought control, coach authoritarian behavior will report higher scores of burnout, resignation, distancing, and unpleasant emotions (anger, anxiety, and dejection).

## Materials and Methods

### Participants

The participants were 268 athletes (*M*_*age* = _ 29.34; *SD* = 12.37; 195 men and 73 women). In the sample, the greatest number of participants were amateur athletes (*n* = 225) and some athletes were professional athletes (*n* = 43). A total of 45 athletes competed at international level, 140 at national level, 260 at regional level and 325 at local level. Moreover, it is important to point out that athletes could compete in more than one level. In terms of hours of practice per week, 59 athletes practiced less than 5 h per week, 95 athletes practiced between 5 and 10 h per week, 62 athletes practiced between 10 and 15 h per week, 37 athletes practiced between 15 and 20 h per week, and 15 athletes practiced more than 20 h per week.

The sample collection was taken following a non-randomized controlled trial, in which the researchers tried to collect participants from all the Spanish regions and ensuring guarantees of ethical guidelines and information collection. Furthermore, to maximize the external validation and replication of the results in different samples, athletes from different sports were included in the present study (30.2% team sport and 69.8% individual sport). As an inclusion criterion, it was selected only competitive athletes from whatever sports (i.e., physical activity practitioners were not allowed to participate in the study). Furthermore, only athletes with coach were selected (those without coach were discarded from the sample).

### Measures

The Leadership Sports Scale (LSS, Chelladurai and Saleh, 1980), validated into Spanish context by Crespo et al. (1994), was used to measure the coach’s leadership. The scale consists of 40 items on a 5-step Likert scale (5 = always, 1 = never). The questionnaire is divided into three versions: (i) the player’s preferred version, in which the behaviors of an ideal coach are described; (ii) player’s perception version, which shows the behaviors of how they perceive their coach; and (iii) the coach’s perception version of their own behavior. In this work, the player’s perception version was used. This scale contains five dimensions: coach autocratic behavior (*a* = *0.82*), social support (*a* = *0.84*), positive feedback (*a* = *0.83*), democratic behavior (*a* = *0.69*), and training and instruction (*a* = *0.92*).

The Sports Emotion Questionnaire (SEQ; Jones et al., 2005) was used to assess the emotions, experienced by athletes in the last competition. The translation into Spanish language was carried out by three independent bilingual translators, who used standardized back-translation procedures (Brislin, 1986). In order to reach the best translated version, the back-translation procedure was repeated interactively until the original and back-translated English versions of the questionnaires were identical. The SEQ is made up by 22 items scored on a 5-point Likert-type scale ranging from 0 (not at all) to 4 (extremely). The scale has five emotions divided in: happiness (4 items; α = 0.88), excitement (4 items; α = 0.76), dejection (5 items; α = 0.91), anxiety (5 items; α = 0.77), and anger (4 items; α = 0.91). Participants completed the SEQ using a 5-point Likert-type scale ranging from 0 (not at all) to 4 (extremely). This scale has indicated good validity and reliability for measuring sport emotions (Allen et al., 2010; Dewar and Kavussanu, 2012). A confirmatory factor analysis was performed with a robust maximum likelihood estimation procedure. Fit indices indicate that the measurement model is acceptable (χ^2^^ =^ 2720.15, *p* < 0.001, CFI = 0.90, RMSEA = 0.05).

The Coping Inventory for Competitive Sport (CICS; Gaudreau and Blondin, 2002) validated into Spanish language by Molinero et al. (2010) is a scale that contains 31 items using a 5-point Likert type scale ranging from 1 (nothing) to 5 (much). It contains 8 factors: resignation (4 items; α = 0.73), relaxation (4 items; α = 0.76), distancing (3 items; α = 0.64), logical analysis (7 items; α = 0.61), seeking support (2 items; α = 0.76), imagery/thought control (5 items; α = 0.68), venting emotions (3 items; α = 0.79), and mental distraction (3 items; α = 0.74).

The Spanish version (Arce et al., 2012) of the Athlete Burnout Questionnaire (ABQ; Raedeke and Smith, 2001) was used to assess burnout in athletes. It contains three subscales measuring emotional/physical exhaustion, sport devaluation, and reduced sense of accomplishment. The scale has 15 items for each dimension with five response options (from 1—almost never to 5—almost ever). Previous researches provided evidence for the content, factorial, construct validity, and reliability of the scores derived from the ABQ (Isoard-Gautheur et al., 2010; Arce et al., 2012). The Cronbach alpha is.82 for emotional/physical exhaustion, 0.64 for reduced accomplishment, and.75 for sport devaluation.

Acquiescence and dishonest participants. The Oviedo scale of infrequency response was used (INF-OV; Fonseca-Pedrero et al., 2009). This is a 12-item self-report measure with a 5-point Likert-type rating scale format (1 totally disagree; 5 totally agree). Its goal is to detect participants who responded randomly, pseudo-randomly or dishonestly on self-reports (e.g., “The distance between Madrid and Barcelona is greater than between Madrid and New York”). The participants with more than four incorrect answers were deleted from the sample. In this study, 10 participants were taken out in the sample.

### Procedure

The research was carried out following international ethical guidelines, and anonymity was preserved. Moreover, the Ethics Committee of Francisco de Vitoria University approved the study. The researchers contacted the Spanish federations (e.g., Table tennis, Tennis, Basketball, CrossFit, Paddle, Football, Volleyball, and Cycling) in order to ask them to announce on their website the requirements to participate in the study. Then, the athletes, who were interested in participating, completed the online survey. Once they accessed to the survey link, they signed an informed consent form, in case participants were children, it was signed by their parents, and after they could begin with the survey questions.

### Data Analyses

The analyses were performed using SPSS 20 version software. Firstly, the data were filtered for multivariate outliers and multicollinearity of scales. Secondly, to increase the confidence in the stability of the cluster solution, a two-step approach including both hierarchical and non-hierarchical cluster analyses were performed using standardized LSS scores (Hair et al., 2010). In particular, to identify the number of clusters (coach leadership profiles), a hierarchical cluster analysis (Ward’s linkage method with squared Euclidian distance) was conducted. Then, a *k* means cluster analysis was performed, using the most appropriate cluster solution identified in stage one. Thirdly, to examine cluster group differences on burnout, coping variables and emotions, a Multivariate analyses of variance (MANOVA) with athletes’ outcome variables (coping, burnout and emotions) entered as the dependent variables, was conducted. In the analyses, to prevent Type I error, a significant multivariate effect (*p* < 0.05) was followed up with subsequent ANOVAs using Bonferroni adjustment (*p* < 0.003 for psychological variables). The Partial eta squared (η^2^) was assessed for providing an index of effect size. Finally, to explore potential demographic clusters confounds, a MANOVA with quantitative demographic variables (age, hours of practice) was conducted to examine cluster group differences on demographic variables. Moreover, a series of chi-square test was conducted with qualitative variables (gender; level of competition (international, national, regional and local); professional versus no professional athletes). Though conducting a series of chi-square test does not allow to assess interacting effects (e.g., gender and level of competition), this analytical explorative analysis was mainly tentative.

## Results

### Coach Leadership Profiles

The agglomeration schedule coefficient and the dendrogram indicated that a two-cluster solution was the most appropriate solution. The non-hierarchical procedure confirmed the hierarchical one as the two clusters were almost identical within the two methods. A MANOVA analysis was conducted to detect significant multivariate effects between the two clusters on the coach leadership dimensions [Wilk’s Lambda = 0.35, *F*(5, 262) = 95.99, *p* < 0.001, η^2^ = 0.64]. In cluster analysis, the *F* tests provided by ANOVA should be used only for descriptive purpose because the clusters have been chosen to maximize the differences among cases in different clusters. Consequently, the observed significance levels should be corrected (Martinent and Ferrand, 2007). However, even in using a conservative significance level of *p* < 0.001, the two clusters were significantly different on all LSS scores (Table 1), thus providing a solid indication for the tenability of a two-cluster solution. Descriptive labels for these clusters are: (a) profile 1 comprising athletes characterized by high scores of training and instruction, authoritarian behavior, social support and positive feedback, and a low score of democratic behavior; and (b) profile 2 comprising athletes reporting low levels of training and instruction, authoritarian behavior, social support and positive feedback and high levels of democratic behavior.

**TABLE 1:** Standardized leadership scale scores across the clusters.

	**Cluster 1 (*n* = 178)**	**Cluster 2 (*n* = 90)**	**F (5.262)**	***P***	**Eta^2^**	**Cronbach *a***
					
	**M (*SD*)**	**M (*SD*)**				
Training and Instruction	0.53 (0.54)	−1.04(0.86)	335.20	0.001^*^	0.55	0.92
Authoritarian Behavior	0.47 (0.72)	−0.94(0.77)	220.78	0.001^*^	0.45	0.82
Democratic Behavior	−0.29(0.88)	0.57 (0.96)	53.95	0.001^*^	0.16	0.69
Social Support	0.49 (0.63)	−0.97(0.87)	247.51	0.001^*^	0.48	0.84
Positive Feedback	0.46 (0.62)	−0.91(0.96)	197.88	0.001^*^	0.42	0.83

^*^p < 0.01.

### Cluster Group Differences on Burnout and Coping Variables

Results of MANOVA (Wilk’s Lambda = 0.83, *F* (16, 25) = 3.05, *p* < 0.001, η^2^ = 0.16) indicated significant differences across clusters on athletes’ psychological outcomes as a whole. In Table 2, Results of ANOVAs showed significant differences (Bonferroni correction, *p* < 0.003) on reduced accomplishment, sports devaluation, happiness and seeking support. In particular, athletes from profile 2 reported significantly (*p* < 0.01) higher scores of reduced accomplishment and sports devaluation and marginally (*p* < 0.05) higher scores of dejection than their counterparts from profile 1. In contrast, athletes from cluster 1 reported significantly higher scores (*p* < 0.01) of seeking support and happiness and marginally (*p* < 0.05) higher scores of logical analysis, imagery/thought control, and excitement.

**TABLE 2:** Cluster Differences on Burnout, Coping, and Emotions.

	**Cluster 1 (*n* = 178)**	**Cluster 2 (*n* = 90)**	**F (16.25)**	***P***	**Eta^2^**	**Cronbach *a***
					
	**M (*SD*)**	**M (*SD*)**				
Emotional/Physical Exhaustion	11.89 (4.22)	11.62 (3.65)	0.28	0.59	0.00	0.82
Reduced Accomplishment	11.46 (3.34)	12.88 (3.49)	10.47	0.001^*^	0.04	0.64
Sport Devaluation	9.11 (3.82)	10.72 (4.08)	10.03	0.002^*^	0.04	0.75
Anxiety	2.00 (0.88)	1.83 (0.90)	2.32	0.12	0.01	0.77
Dejection	0.59 (0.77)	0.83 (0.92)	4.93	0.02	0.02	0.91
Excitement	3.10 (0.66)	2.83 (0.80)	8.64	0.004	0.03	0.76
Happiness	3.28 (0.69)	2.89 (0.77)	16.98	0.001^*^	0.06	0.88
Anger	0.60 (0.85)	0.77 (0.97)	2.13	0.14	0.01	0.91
Resignation	7.66 (2.97)	8.24 (3.40)	2.06	0.15	0.01	0.73
Relaxation	13.73 (3.15)	13.16 (2.90)	2.00	0.15	0.01	0.76
Distancing	6.94 (2.18)	6.73 (1.99)	0.62	0.43	0.00	0.64
Logical Analysis	25.52 (3.97)	24.23 (3.74)	6.52	0.01	0.02	0.61
Seeking Support	7.66 (1.84)	6.45 (2.14)	23.16	0.001^*^	0.08	0.76
Imagery/Thought Control	18.87 (3.36)	17.86 (3.03)	5.73	0.017	0.02	0.68
Venting Emotions	8.22 (3.00)	8.12 (2.85)	0.07	0.79	0.00	0.79
Mental Distraction	6.52 (2.74)	6.63 (2.54)	0.10	0.75	0.00	0.74

^*^p < 0.01.

### Cluster Group Differences on Demographic Variables

Results of chi square tests showed no significant difference (*p* > 0.05) across gender [χ^2^(1) = 0.19] practice level [χ^2^(4) = 0.13; *p* > 0.05], successes [χ^2^(4) = 0.41; *p* > 0.05] and professional versus no professional athletes [χ^2^(1) = 0.74; *p* > 0.05]. Moreover, results of MANOVA [Wilk’s Lambda = 0.98, *F*(2) = 265, *p* > 0.05; η^2^ = 0.002] showed no significant differences in age and hours of practice across the two clusters.

## Discussion

The goals of the study were to identify coach leadership profiles and examine whether participants from such profiles significantly differed on burnout, emotions and coping. The results obtained in the present study furthered knowledge base on coach leadership in sport in two ways. Firstly, the cluster analysis approach provided a parsimonious and meaningful way to summarize coach leadership dimensions perceived by athletes (rather than to individually consider the several coach leadership dimensions). Secondly, the cluster methodology, used in the present study, highlighted the relationships of perceived coach leadership profiles with a variety of athletes’ outcomes (i.e., coping, burnout and competitive emotions). Previous studies, on the topic of coach leadership, neglected the multivariate nature of the construct, whereas a profile approach, could make sense because the different coach leadership dimensions can co-occur and co-exist in sports context.

Two coach leadership profiles emerged from the cluster analyses: (a) profile 1 characterized by high scores of training and instruction, authoritarian behavior, social support and positive feedback, and low scoresof democratic behavior; and (b) profile 2 comprising athletes reporting low levels of training and instruction, authoritarian behavior, social support, and positive feedback and high levels of democratic behavior. These findings highlighted the variability of behaviors that a coach might use in sport context and provided evidence that distinct coach behaviors can co-occur from the point of view of the athletes. It is also noteworthy that the naturally occurring combinations of the dimensions of coach leadership emerging from the cluster analyses (profile 1) were rather surprising. In particular, athletes from the profile 1 reported simultaneously high scores on authoritarian behavior, social support, and positive feedbacks, whereas previous studies showed that authoritarian behavior were consistently related to negative athletes’ outcomes (athlete burnout) but social support and positive feedbacks were consistently related to positive athletes’ outcomes (well-being, fun, motivation, cohesion) (Hollembeak and Amorose, 2005; Dell’Antonio and Couto, 2014; Sullah et al., 2014; Cruz and Kim, 2017). Moreover, athletes from the profile 2 reported high scores on democratic behavior and low scores on social support and positive feedbacks whereas previous studies showed that these three dimensions of coach leadership were consistently related to positive athletes’ outcomes (Hollembeak and Amorose, 2005; Sullah et al., 2014; Cruz and Kim, 2017). As a whole, these results provided new insights on the coach leadership literature in sports in highlighting the naturally occurring combinations of coach leadership dimensions perceived by competitive athletes.

Apart from offering a description of naturally occurring combinations of coach leadership dimensions perceived by athletes, this study examined the relationship between coach leadership profiles and athletes’ coping, emotions and burnout. In particular, results indicated that athletes from the profile 2 reported significantly higher scores of reduced accomplishmen and sports devaluation, marginally higher scores of dejection as well as significantly lower scores of happiness, seeking support and marginally lower scores of excitement, logical analysis, and imagery/thought control. In other words, results of the present study suggested that athletes from the profile 1 were characterized by the best psychological adjustment as inferred by the athletes’ scores of burnout, coping and emotion. The cluster results highlighted the potential benefits of considering multiple dimensions of coach leadership simultaneously when examining the functional nature of this construct. Although previous studies provided compelling evidence that democratic behavior is related with positive athletes’ outcomes (Ryan and Deci, 2000; Gillet et al., 2010), results of the present study highlighted that a profile characterized by high scores of democratic behavior in combination with low scores of training and instruction, positive feedback and social support is related with negative athletes’ outcomes (i.e., sport devaluation, reduced accomplishment, dejection). Similarly, whereas previous studies provided compelling evidence that authoritarian behavior is related with negative athletes’ outcomes (Ryan and Deci, 2000; Gillet et al., 2010; Cruz and Kim, 2017), results of the present study highlighted that a profile, characterized by high scores of authoritarian behavior in combination with high scores of training and instruction, positive feedback and social support is related with positive athletes’ outcomes (i.e., happiness, excitement, logical analysis, imagery/thought control). Thus, differences were observed in the results obtained from the person- (cluster analysis) and variable-centered (multiple regression or correlational analysis) approaches. As a whole, results of the present study and of previous studies provided evidence that a single coach leadership dimension is most likely operating in conjunction with other coach leadership dimension. Thus, much of the information could be lost when coach leadership dimensions are examined independently with a variable-centered approach. As such, further research should adopt a coach leadership profile approach to explore the functional nature of the construct in naturalistic sports settings.

A notable limit of cluster analytic studies is the data-driven approach in determining the combination and the number of profiles (Martinent et al., 2013). Thus, future research should replicate the present results with athletes from distinct ages, cultures, or practice levels as a mean to demonstrate the tenability of coach leadership profiles. It could also be particularly fruitful to conduct a longitudinal analysis of coach leadership profiles in order to explore the change and stability of coach leadership profiles at intra-individual and inter-individual levels. Moreover, common method bias might have distorted the findings of the present study as all the study variables were measured using a single source of data (self-report questionnaires). Hence, future research should complement self-reported data with objective ratings (performance scores) or peer-rating (coach perceptions).

Other limitations are sample size and the non-randomized controlled trial design. Regarding sample, it could be big enough taking into account the huge amount number of athletes, for that reason the study should be replicated with a bigger sample to ensure that results evolve in the same way. Concerning non-randomized controlled trial design, it is tough to collect a big amount of sample following randomized designs for the difficulty to find athletes due to its demanding time tables and competitions schedule.

Notwithstanding these limits, the present study proposed an alternative person-centered approach that may provide researchers and practitioners with a useful way to examine naturally occurring combinations of the coach leadership dimensions. Furthermore, the combination of exploratory and confirmatory data analyses and this alternative of a person-centered approach, should be considered as a strength of the study. In particular, findings of the present study highlighted that the coach leadership profiles allow examining the construct of coach leadership within a holistic approach, teasing out the complex associations with key athlete outcomes, such as burnout, coping, and emotion. Conducting person-centered analysis using a cluster analytic approach may be a fruitful avenue for research investigating coach leadership in sports settings, as well as in leadership research in general.

## Data Availability

The data of the study can be found by contacting the corresponding author.

## Ethics Statement

This study was approved by the ethics committee of Francisco de Vitoria University. Furthermore, anonymity was preserved and it follows APA ethical guidelines. Moreover, this study only used self-report measures (questionnaires).

## Author Contributions

HG-G collected the sample, and wrote the sections “Introduction” and “Discussion”. GM conducted the data analysis and reviewed the whole manuscript. ATM helped in the sample collection and reviewed the writing of the whole manuscript.

## Conflict of Interest Statement

The authors declare that the research was conducted in the absence of any commercial or financial relationships that could be construed as a potential conflict of interest.
